# Genome mining reveals abiotic stress resistance genes in plant genomes acquired from microbes *via* HGT

**DOI:** 10.3389/fpls.2022.1025122

**Published:** 2022-11-02

**Authors:** Liangzhi Li, Shuguang Peng, Zhenhua Wang, Teng Zhang, Hongguang Li, Yansong Xiao, Jingjun Li, Yongjun Liu, Huaqun Yin

**Affiliations:** ^1^ School of Minerals Processing and Bioengineering, Central South University, Changsha, China; ^2^ Key Laboratory of Biometallurgy of Ministry of Education, Central South University, Changsha, China; ^3^ Hunan Tobacco Science Institute, Changsha, China; ^4^ Zhangjiajie Tobacco Company of Hunan Province, Zhangjiajie, China; ^5^ Hunan Urban and Rural Environmental Construction Co., Ltd, Changsha, China; ^6^ Chenzhou Tobacco Company of Hunan Province, Chenzhou, China

**Keywords:** abiotic stress resistance, plant, microbe, horizontal gene transfer, phylogeny

## Abstract

Colonization by beneficial microbes can enhance plant tolerance to abiotic stresses. However, there are still many unknown fields regarding the beneficial plant-microbe interactions. In this study, we have assessed the amount or impact of horizontal gene transfer (HGT)-derived genes in plants that have potentials to confer abiotic stress resistance. We have identified a total of 235 gene entries in fourteen high-quality plant genomes belonging to phyla *Chlorophyta* and *Streptophyta* that confer resistance against a wide range of abiotic pressures acquired from microbes through independent HGTs. These genes encode proteins contributed to toxic metal resistance (e.g., ChrA, CopA, CorA), osmotic and drought stress resistance (e.g., Na^+^/proline symporter, potassium/proton antiporter), acid resistance (e.g., PcxA, ArcA, YhdG), heat and cold stress resistance (e.g., DnaJ, Hsp20, CspA), oxidative stress resistance (e.g., GST, PoxA, glutaredoxin), DNA damage resistance (e.g., Rad25, Rad51, UvrD), and organic pollutant resistance (e.g., CytP450, laccase, CbbY). Phylogenetic analyses have supported the HGT inferences as the plant lineages are all clustering closely with distant microbial lineages. Deep-learning-based protein structure prediction and analyses, in combination with expression assessment based on codon adaption index (CAI) further corroborated the functionality and expressivity of the HGT genes in plant genomes. A case-study applying fold comparison and molecular dynamics (MD) of the HGT-driven CytP450 gave a more detailed illustration on the resemblance and evolutionary linkage between the plant recipient and microbial donor sequences. Together, the microbe-originated HGT genes identified in plant genomes and their participation in abiotic pressures resistance indicate a more profound impact of HGT on the adaptive evolution of plants.

## Introduction

Plants, the most profoundly distributed lineage on earth, possess the remarkable abilities to convert solar energy into bio-matter and produce oxygen while consume the greenhouse gas carbon dioxide. At the same time, plants have to face the frequent offense of inhospitable abiotic stresses caused by the constant fluctuations of global climate since plants cannot “run away” from them. The challenging conditions like drought, radiation, nutrient deficiencies, temperature extremes (cold and heat), metal ion toxicity, salinity and organic pollution are typical abiotic stress factors that plants frequently encounter (Zhang et al., 2022). These abiotic factors have drastically influenced plant distribution and growth status globally (Nolan et al., 2018). It is long known that beneficial microbes are helping plants to overcome climate change and other adverse conditions through a variety of ways (Schützendübel and Polle, 2002; Haney et al., 2015; Murali et al., 2021). For example, microbes can increase the activity of plant antioxidant defense enzyme (Mastouri et al., 2012), release beneficial volatile organic compounds (VOCs) that trigger resistance response (Liu and Zhang, 2015), and modulate the expression of plant metal transporters against the accumulation of heavy metals (Zhou et al., 2020). However, many aspects of the plant-microbe interactions for mutual benefits are still in the “black box” and lack in-depth mechanism analysis.

Horizontal gene transfer (HGT), referring to the exchange of genetic material between distant lineages, is considered as an effective mechanism to spread evolutionary success in both prokaryotes and eukaryotes (Huang, 2013; Daubin and Szöllősi, 2016). Studies on HGT genes introduced into eukaryotic taxa have been expanding fast in the last twenty years as next-generation sequencing technology has made large-scale genome data available (Soucy et al., 2015). Recent researches have demonstrated that genes in plant genomes important for the function of specific plant activities, including the biosynthesis of plant structure components and hormones, plant defense, nitrogen recycling and stress resistance, can be obtained *via* the omnipresent HGT process (Yue et al., 2012). It is strongly proposed in a recent review that HGTs possess the potency to drastically influence plant evolutionary path by imparting selective advantages (Wickell and Li, 2020). Here are some examples illustrating the importance of microbe-derived HGTs in plants that confer adaptive benefits. Firstly, it is found that the ancestor of land plants acquired a phenylalanine ammonia lyase (PAL), the entry point of the phenylpropanoid pathway, through HGT during symbioses with soil bacteria and fungi (Emiliani et al., 2009); The second case is that *Physcomitrella patens* is suggested to acquire from bacteria acyl-activating enzyme 18 (AAE18)/flavin monooxygenase (YUC3) for auxin biosynthesis, guanine deaminase, allantoate amidohydrolase and ureidoglycolate amidohydrolase associated with purine degradation, as well as subtilases for protein degradation (Yue et al., 2012). Besides, Li et al. (2018) detected that the squalene-hopene cyclase (SHC) encoding genes in three plant clades (mosses, liverworts and lycophytes/ferns) are interspersing with bacterial SHCs, suggesting the occurrence of independent and repeated HGT events for enhanced adaption since the product of SHC, hopanoid, constitutes plant cell membrane important for maintaining cell integrity. Lastly, it is reported that the adaptive genes in *Zygnematophyceae*, GRAS and PYR/PYL/RCAR that enhance resistance to abiotic stresses (e.g., desiccation), were obtained through HGT from nearby soil bacteria (Cheng et al., 2019). However, many of these previous investigations focused mainly on individual plant lineage or limited HGT events on important economic aspects such as the valuable plant metabolism pathways. Despite these efforts, we still have little knowledge on the amount or impact of HGT-derived genes in plants that have potentials to confer resistance against a wide range of abiotic stresses (e.g., low pH, osmotic pressure, heat and cold stress) and hence promote the growth of plants in extreme conditions. It is still an open question that if there is microbe-originated HGT(s) that has facilitated plant to adapt such abiotic stresses. And if there are such HGT genes, what are their functions in combating abiotic stresses and to what extent are they distributed in the plant genomes?

Against such background, in this study, we have chosen fourteen high-quality plant genomes covering major plant taxa (Clade *Viridiplantae*) within Phyla *Chlorophyta* and *Streptophyta*, and tried to uncover and scrutinize putative microbe-originated HGT genes that are associated with abiotic stress resistance in these genomes employing a combination of methods previously showed to be highly effective in identifying HGT between distantly related species (Schönknecht et al., 2014), including BLASTP-based searches and gene tree reconciliation. To further testify the expression potential of the detected HGT genes, we supply codon adaption index calculation, deep-learning-driven protein structure prediction, fold alignment, and molecular dynamics to assess the expression potential and functionality of the abiotic stress resistance genes acquired by plants from microbial lineages. Our findings indicated that the HGT events in beneficial microbes putatively colonizing plant might be an important source of acquisitions of genes that help enhance plant tolerance against abiotic stresses. This study has also advanced our understanding on plant-microbe interactions and the adaptive evolution of plants to encounter adverse conditions.

## Results

### General information

Detailed information of the fourteen high-quality eukaryotic plant genomes covering major plant taxa (clade *Viridiplantae*) within phyla *Chlorophyta* and *Streptophyta* chosen for downstream analyses is provided in **Table 1**. Putative microbe-originated HGT genes in these genomes employing the BLASTP-based IMGAP pipeline (Markowitz et al., 2010), followed by manual pickup of microbe-originated genes that are associated with abiotic stress resistance. These processes eventually produce a total of 235 gene entries, as listed in **Table 2** and **Supplementary Tables S1A** and **S1B** at https://doi.org/10.6084/m9.figshare.20530083.v1. Detailed descriptions are provided in the following subsections.

**Table 1:** High-quality eukaryotic plant genomes covering major plant taxa (clade *Viridiplantae*) within phyla *Chlorophyta* and *Streptophyta* chosen for microbe-originated horizontal gene transfer (HGT) detection in this study.

Genome name	Phylum	NCBI bioproject	NCBI genbank ID	IMG genome ID	Sequencing depth	Sequencing quality	Sequencing status	Genome size (bp)	Scaffold count	Gene count	GC %
*Chlamydomonas reinhardtii* CC-503 cw92 mt+	*Chlorophyta*	PRJNA12260	ABCN00000000	649410502	10x	Level 2: High-Quality Draft	Complete	120404952	1558	14546	63.87
*Volvox carteri* f. nagariensis 69-1b	*Chlorophyta*	PRJNA13109	ACJH00000000	2507525017	8x	Level 2: High-Quality Draft	Complete	137835555	1265	14542	55.97
*Chlorella variabilis* NC64A	*Chlorophyta*	PRJNA45853	ADIC00000000	2507525016	8.9x	Level 1: Standard Draft	Complete	46159515	414	9791	67.14
*Arabidopsis thaliana* Columbia	*Streptophyta*	PRJNA13190	GCA_000211275	639560100		Level 6: Finished	Complete	119707899	7	31370	36.06
*Physcomitrella patens* patens (Moss)	*Streptophyta*	PRJNA13064	ABEU00000000	649410504	8.63x	Level 2: High-Quality Draft	Complete	477845029	1992	35938	33.58
*Glycine max* cultivar Williams 82 (Soybean)	*Streptophyta*	PRJNA19861	ACUP00000000	2507525014	8.5x	Level 2: High-Quality Draft	Complete	973344380	1168	55787	34.75
*Populus balsamifera* trichocarpa (Balsam poplar)	*Streptophyta*	PRJNA10772	AARH00000000	649410505	7.5x	Level 2: High-Quality Draft	Complete	480871302	20644	40566	33.55
*Zea mays mays* cv. B73 (Maize)	*Streptophyta*	PRJNA10769	CM000786, CM000777, GK000031	2507525013	6x	Level 6: Finished	Complete	2065722704	11	106044	46.89
*Arabidopsis lyrata* lyrata MN47	*Streptophyta*	PRJNA41137	ADBK00000000	649410501	8x	Level 2: High-Quality Draft	Complete	206667935	695	32549	36.08
*Vitis vinifera* PN40024 (Grape Vines)	*Streptophyta*	PRJNA34679	CAAP00000000	649410508	12x	Level 2: High-Quality Draft	Complete	791124756	3535	24954	34.51
*Ricinus communis* Hale (Castor bean)	*Streptophyta*	PRJNA16585	AASG00000000	649410506	2.45x	Level 2: High-Quality Draft	Complete	350458699	25762	31894	33.84
*Sorghum bicolor* BTx623	*Streptophyta*	PRJNA13876	ABXC00000000	2507525011	4x	Level 2: High-Quality Draft	Complete	738540932	3304	29448	43.93
*Solanum tuberosum* DM 1-3 516 R44 (Potato)	*Streptophyta*	PRJNA63145	AEWC00000000	2507525040	114x	Level 2: High-Quality Draft	Complete	727424546	66254	24377	34.8
*Fragaria vesca Hawaii* 4 (Woodland strawberry)	*Streptophyta*	PRJNA60037	AEMH00000000	2507525039	49x	Level 2: High-Quality Draft	Complete	214219504	3254	7931	38.4

**Table 2:** Microbe-originated HGT genes identified in tested plant genomes that associated with miscellaneous abiotic stress resistance.

Plant recipient	Recipient gene accession (IMG)	Gene function	Donor gene accession (IMG)	Microbial donor	Donor taxon
Metal resistance					
*Chlorella variabilis NC64A*	2507982899	Magnesium-translocating P-type ATPase MgtA	646357403	*Escherichia coli O111:H 11128*	*Bacteria; Proteobacteria*
*Chlamydomonas reinhardtii CC-503 cw92 mt+*	649485460	Magnesium and cobalt transport protein	2508406796	*Spizellomyces punctatus DAOM BR117*	*Eukaryota; Chytridiomycota*
*Chlamydomonas reinhardtii CC-503 cw92 mt+*	649493513	Copper homeostasis protein CutC	2506487166	*Emticicia oligotrophica GPTSA100-15*	*Bacteria; FCB group*
*Physcomitrella patens patens*	649531133	Copper resistance protein CopC	648722241	*Paenibacillus curdlanolyticus YK9*	*Bacteria; Firmicutes*
*Ricinus communis Hale*	649585425	Chromate transporter ChrA	649771835	*Methylovorus* sp. *MP688*	*Bacteria; Proteobacteria*
*Arabidopsis thaliana Columbia*	639572405	Copper-transporting ATPase	645867480	*Talaromyces marneffei ATCC 18224*	*Eukaryota; Ascomycota*
*Arabidopsis thaliana Columbia*	639588060	P-type copper ATPase	645729519	*Aspergillus clavatus NRRL 1*	*Eukaryota; Ascomycota*
*Volvox carteri f. nagariensis 69-1b*	2507995811	Magnesium and cobalt transport protein	2508406796	*Spizellomyces punctatus DAOM BR117*	*Eukaryota; Chytridiomycota*
*Vitis vinifera PN40024*	649546378	Ca^2+^-transporting atpase	2509695751	*Schizophyllum commune H4-8*	*Eukaryota; Opisthokonta; Fungi*
*Physcomitrella patens patens*	649501412	Acid phosphatase	2509205796	*Batrachochytrium dendrobatidis JEL423*	*Eukaryota; Chytridiomycota; Chytridiomycetes*
Osmotic and drought stress resistance					
*Physcomitrella patens patens*	649496502	Choline dehydrogenase BetA	2509380569	*Ensifer aridi TW10*	*Bacteria; Proteobacteria; Deltaproteobacteria*
*Volvox carteri f. nagariensis 69-1b*	2508002683	Chloride intracellular channel 6	639857298	*Stigmatella aurantiaca DW4/3-1*	*Bacteria; Proteobacteria; Deltaproteobacteria*
*Volvox carteri f. nagariensis 69-1b*	2507995432	Potassium/proton antiporter	645972328	*Streptomyces* sp. *SPB78*	*Bacteria; Actinobacteria; Actinomycetia*
*Chlorella variabilis NC64A*	2507981403	Sarcosine oxidase SoxA	646435156	*Sphaerobacter thermophilus 4ac11, DSM 20745*	*Bacteria; Chloroflexi; Thermomicrobia*
*Chlorella variabilis NC64A*	2507981739	Na^+^/proline symporter	2508394518	*Allomyces macrogynus ATCC 38327 (fungus)*	*Eukaryota; Blastocladiomycota; Blastocladiomycetes*
*Chlorella variabilis NC64A*	2507982753	Chloride channel protein ClcA	637771792	*Synechococcus* sp. *CC9902*	*Bacteria; Cyanobacteria; Synechococcales*
*Arabidopsis thaliana Columbia*	639580571	Na^+^/solute symporter	646259962	*Oxalobacter formigenes HOxBLS*	*Bacteria; Proteobacteria; Betaproteobacteria*
*Arabidopsis thaliana Columbia*	639566001	Na/K ATPase alpha 1 subunit, putative	645866880	*Talaromyces marneffei ATCC 18224*	*Eukaryota; Ascomycota; Eurotiomycetes*
*Chlamydomonas reinhardtii CC-503 cw92 mt+*	649489365	Sodium/calcium exchanger	649408713	*Perkinsus marinus PmCV4CB5 2B3 D4*	*Eukaryota; Perkinsozoa; Perkinsida*
*Chlamydomonas reinhardtii CC-503 cw92 mt+*	649487367	Sodium/proton antiporte, CPA1	637235733	*Nostoc* sp. *PCC 7120*	*Bacteria; Cyanobacteria; Nostocales*
*Chlamydomonas reinhardtii CC-503 cw92 mt+*	649492246	Zinc-nutrition responsive transporter, ZIP family	649337865	*Phaeodactylum tricornutum CCAP1055/1*	*Eukaryota; Bacillariophyta; Bacillariophyceae*
*Glycine max cultivar Williams 82*	2507918029	Maltooligosyl trehalose hydrolase (EC 3.2.1.141)	651457131	*Xanthomonas vesicatoria Maraite, ATCC 35937*	*Bacteria; Proteobacteria; Gammaproteobacteria*
*Arabidopsis thaliana Columbia*	639571770	Trehalose 6-phosphate synthase (EC 2.4.1.15)	646433456	*Thermanaerovibrio acidaminovorans Su883, DSM 6589*	*Bacteria; Synergistetes; Synergistia*
*Arabidopsis thaliana Columbia*	639573047	Teichoic acid biosynthesis protein B	646070519	*Staphylococcus aureus A9635*	*Bacteria; Firmicutes; Bacilli*
*Chlamydomonas reinhardtii CC-503 cw92 mt+*	649484689	Cellulose synthase (UDP-forming)	637692951	*Cupriavidus pinatubonensis JMP134*	*Bacteria; Proteobacteria; Betaproteobacteria*
*Chlorella variabilis NC64A*	2507980628	dTDP-D-glucose 4,6-dehydratase RmlB	641205904	*Acanthocystis turfacea Chlorella virus 1*	*Viruses; dsDNA viruses, no RNA stage; Megaviricetes*
*Physcomitrella patens patens*	649498952	Capsular exopolysaccharide family	2505768802	*Fischerella thermalis JSC-11*	*Bacteria; Cyanobacteria; Synechococcales*
*Chlorella variabilis NC64A*	2507978560	Malate synthase (EC 2.3.3.9)	641353162	*Sorangium cellulosum So ce 56*	*Bacteria; Proteobacteria; Deltaproteobacteria*
*Chlorella variabilis NC64A*	2507978803	Malate synthase (EC 2.3.3.9)	641353162	*Sorangium cellulosum So ce 56*	*Bacteria; Proteobacteria; Deltaproteobacteria*
*Chlorella variabilis NC64A*	2507978611	Isocitrate lyase (EC 4.1.3.1)	643430771	*Anoxybacillus flavithermus WK1, DSM 2641*	*Bacteria; Firmicutes; Bacilli*
*Physcomitrella patens patens*	649526838	Transaldolase/Fructose-6-phosphate aldolase	647578161	*Synechococcus* sp. *PCC 7335*	*Bacteria; Cyanobacteria; Synechococcales*
Heat and cold stress					
*Glycine max cultivar Williams 82*	2507922078	Stromal 70 kDa chaperone protein DnaK	643477038	*Rippkaea orientalis PCC 8801*	*Bacteria; Cyanobacteria; Chroococcales*
*Chlorella variabilis NC64A*	2507984527	DnaJ-class molecular chaperone with C-terminal Zn finger domain	645858879	*Pyrenophora tritici-repentis Pt-1C-BFP*	*Eukaryota; Ascomycota; Dothideomycetes*
*Chlorella variabilis NC64A*	2507978787	DNA-binding protein HU	644676974	*Bacillus thuringiensis sv. monterrey BGSC 4AJ1*	*Bacteria; Firmicutes; Bacilli*
*Chlorella variabilis NC64A*	2507982059	DnaJ-class molecular chaperone with C-terminal Zn finger domain	2507660422	*Phytophthora ramorum Pr102, UCD Pr4*	*Eukaryota; Oomycota; Peronosporales*
*Chlorella variabilis NC64A*	2507982566	Chaperonin GroEL (HSP60 family)	2509191772	*Ectocarpus siliculosus Ec 32 (CCAP 1310/04)*	*Eukaryota; Phaeophyceae; Ectocarpales*
*Chlorella variabilis NC64A*	2507983822	Small heat shock protein (HSP20) family	641146850	*Marinobacter algicola DG893*	*Bacteria; Proteobacteria; Gammaproteobacteria*
*Chlorella variabilis NC64A*	2507983994	DnaJ molecular chaperone homology domain	2507660422	*Phytophthora ramorum Pr102, UCD Pr4*	*Eukaryota; Oomycota; Peronosporales*
*Chlorella variabilis NC64A*	2507984993	Heat shock protein 70	2509695036	*Schizophyllum commune H4-8*	*Eukaryota; Opisthokonta; Fungi*
*Volvox carteri f. nagariensis 69-1b*	2507993038	DnaJ-class molecular chaperone with C-terminal Zn finger domain	642750380	*Hydrogenobaculum* sp. *Y04AAS1*	*Bacteria; Aquificae; Aquificae*
*Fragaria vesca Hawaii 4*	2508472453	Heat shock protein 70KD	638270255	*Guillardia theta*	*Eukaryota; Cryptophyceae; Pyrenomonadales*
*Arabidopsis thaliana Columbia*	639560148	Heat shock protein. Metallo peptidase. MEROPS family M48B	639002844	*Nitrococcus mobilis Nb-231*	*Bacteria; Proteobacteria; Gammaproteobacteria*
*Chlamydomonas reinhardtii CC-503 cw92 mt+*	649482610	Heat shock factor binding protein 1	639628046	*Trypanosoma brucei brucei 927/4 GUTat10.1*	*Eukaryota; Euglenozoa; Kinetoplastea*
*Ricinus communis Hale*	649563104	Chaperone clpb, heat shock protein	637065254	*Escherichia coli O157:H7 EDL933 (EHEC)*	*Bacteria; Proteobacteria; Gammaproteobacteria*
*Ricinus communis Hale*	649583866	Molecular chaperone	2509190845	*Ectocarpus siliculosus Ec 32 (CCAP 1310/04)*	*Eukaryota; Phaeophyceae; Ectocarpales*
*Ricinus communis Hale*	649584950	Cold-shock DNA-binding protein CspA	642599004	*Paraburkholderia phymatum STM815*	*Bacteria; Proteobacteria; Betaproteobacteria*
*Volvox carteri f. nagariensis 69-1b*	2507995359	Chitinase	650773356	*Burkholderia gladioli BSR3*	*Bacteria; Proteobacteria; Betaproteobacteria*
*Chlamydomonas reinhardtii CC-503 cw92 mt+*	649491057	Chitinase family 18 (EC:3.2.1.14)	637922967	*Saccharophagus degradans 2-40*	*Bacteria; Proteobacteria; Gammaproteobacteria*
*Chlamydomonas reinhardtii CC-503 cw92 mt+*	649492930	Chitinase	2508410311	*Spizellomyces punctatus DAOM BR117*	*Eukaryota; Chytridiomycota; Chytridiomycetes*
*Ricinus communis Hale*	649570195	Ribulose 1,5-bisphosphate carboxylase large subunit (EC 4.1.1.39)	647109286	*Raphidiopsis brookii D9*	*Bacteria; Cyanobacteria; Nostocales*
*Chlamydomonas reinhardtii CC-503 cw92 mt+*	649488017	Mitochondrial carbonic anhydrase, beta type	648186377	*Gloeothece verrucosa PCC 7822*	*Bacteria; Cyanobacteria; Chroococcales*
*Chlamydomonas reinhardtii CC-503 cw92 mt+*	649410718	Photosystem II protein VI	648390068	*Limnospira indica PCC 8005*	*Bacteria; Cyanobacteria; Oscillatoriales*
*Chlamydomonas reinhardtii CC-503 cw92 mt+*	649410720	Photosystem II cytochrome *b6/f* complex subunit V	637799911	*Synechococcus elongatus PCC 7942*	*Bacteria; Cyanobacteria; Synechococcales*
Oxidative stress resistance					
*Volvox carteri f. nagariensis 69-1b*	2507998527	Peroxin-13	645816130	*Coprinopsis cinerea okayama7#130*	*Eukaryota; Basidiomycota; Agaricomycetes*
*Volvox carteri f. nagariensis 69-1b*	2507998720	Haem peroxidase	2506751490	*Thiothrix nivea JP2, DSM 5205*	*Bacteria; Proteobacteria; Gammaproteobacteria*
*Chlorella variabilis NC64A*	2507978793	Glutathione S-transferase	2504722272	*Pseudomonas fluorescens HK44*	*Bacteria; Proteobacteria; Gammaproteobacteria*
*Chlorella variabilis NC64A*	2507980056	Peroxiredoxin Q/BCP	645811546	*Coprinopsis cinerea okayama7#130*	*Eukaryota; Basidiomycota; Agaricomycetes*
*Physcomitrella patens patens*	649527966	Cysteine synthase A	640878576	*Parvibaculum lavamentivorans DS-1*	*Bacteria; Proteobacteria; Alphaproteobacteria*
*Chlorella variabilis NC64A*	2507979706	Glutaredoxin	2509193249	*Ectocarpus siliculosus Ec 32 (CCAP 1310/04)*	*Eukaryota; Phaeophyceae; Ectocarpales*
*Chlamydomonas reinhardtii CC-503 cw92 mt+*	649493089	Glutaredoxin, CGFS type	2508453811	*Saprolegnia parasitica CBS 223.65*	*Eukaryota; Oomycota; Saprolegniales*
*Physcomitrella patens patens*	649517225	L-glutamine synthetase (EC 6.3.1.2)	2505791185	*Runella slithyformis LSU4, DSM 19594*	*Bacteria; FCB group; Bacteroidetes*
*Volvox carteri f. nagariensis 69-1b*	2507994380	Homocysteine/selenocysteine methylase (S-methylmethionine-dependent)	651485911	*Rubrivivax benzoatilyticus JA2*	*Bacteria; Proteobacteria; Betaproteobacteria*
*Zea mays mays* cv. *B73*	2507869489	Glutamate synthase (NADPH/NADH)	649510728	*Physcomitrella patens patens*	*Eukaryota; Streptophyta; Bryopsida*
*Glycine max cultivar Williams 82*	2507946040	Glutamate synthase (NADH) large subunit (EC 1.4.1.14)	638941273	*Vibrio* sp. *MED222*	*Bacteria; Proteobacteria; Gammaproteobacteria*
*Volvox carteri f. nagariensis 69-1b*	2507995895	Folylpolyglutamate synthase	2508319981	*Micromonas pusilla NOUM17, RCC 299*	*Eukaryota; Chlorophyta; Mamiellophyceae*
*Arabidopsis thaliana Columbia*	649593200	Glutamate synthase (ferredoxin) (EC 1.4.7.1)	637875655	*Synechococcus* sp. *JA-2-3B’a(2-13)*	*Bacteria; Cyanobacteria; Synechococcales*
*Arabidopsis thaliana Columbia*	649604117	Glutamate synthase (ferredoxin) (EC 1.4.7.1)	640085243	*Prochlorococcus marinus NATL1A*	*Bacteria; Cyanobacteria; Synechococcales*
*Chlamydomonas reinhardtii CC-503 cw92 mt+*	649485827	Thiol-disulfide isomerase and thioredoxins	2508407653	*Spizellomyces punctatus DAOM BR117*	*Eukaryota; Chytridiomycota; Chytridiomycetes*
*Populus balsamifera trichocarpa*	649557489	Cytochrome-c oxidase	645666500	*Talaromyces marneffei ATCC 18224*	*Eukaryota; Ascomycota; Eurotiomycetes*
*Ricinus communis Hale*	649585270	Electron transfer flavoprotein beta subunit	646832083	*Methylotenera versatilis 301, JCM 17579*	*Bacteria; Proteobacteria; Betaproteobacteria*
*Volvox carteri f. nagariensis 69-1b*	2507995502	Ubiquinol-cytochrome *c* reductase cytochrome *b* subunit	640951028	*Rickettsia canadensis McKiel*	*Bacteria; Proteobacteria; Alphaproteobacteria*
*Volvox carteri f. nagariensis 69-1b*	2508002619	Menaquinol-cytochrome c reductase iron-sulfur subunit precursor	639801009	*Paenarthrobacter aurescens TC1*	*Bacteria; Actinobacteria; Actinomycetia*
*Physcomitrella patens patens*	649529911	*c*-type cytochrome synthesis protein Ycf5	641534169	*Microcystis aeruginosa NIES-843*	*Bacteria; Cyanobacteria; Chroococcales*
*Arabidopsis thaliana Columbia*	639588215	Cytochrome c oxidase assembly protein	639887067	*Synechococcus* sp. *BL107*	*Bacteria; Cyanobacteria; Synechococcales*
*Chlorella variabilis NC64A*	2507979399	Phytoene dehydrogenase	641347970	*Sorangium cellulosum So ce 56*	*Bacteria; Proteobacteria; Deltaproteobacteria*
*Arabidopsis thaliana Columbia*	639581518	Isocitrate dehydrogenase, NADP-dependent	637833532	*Hahella chejuensis KCTC 2396*	*Bacteria; Proteobacteria; Gammaproteobacteria*
*Chlorella variabilis NC64A*	2507984961	Thiamine biosynthesis protein ThiC	2509194402	*Ectocarpus siliculosus Ec 32 (CCAP 1310/04)*	*Eukaryota; Phaeophyceae; Ectocarpales*
*Volvox carteri f. nagariensis 69-1b*	2508000640	Thiamine pyrophosphate enzyme	651583914	*Sphingomonas* sp. *S17*	*Bacteria; Proteobacteria; Alphaproteobacteria*
*Arabidopsis thaliana Columbia*	639576678	Thiamine pyrophosphate protein	646441210	*Thermobaculum terrenum YNP1, ATCC BAA-798*	*Bacteria; Chloroflexi; Thermobaculum*
*Chlorella variabilis NC64A*	2507979588	Thiamine monophosphate synthase	2502685596	*Pontibacter actiniarum DSM 19842*	*Bacteria; FCB group; Bacteroidetes*
*Chlorella variabilis NC64A*	2507980977	Selenocysteine lyase	646501278	*Conexibacter woesei ID131577, DSM 14684*	*Bacteria; Actinobacteria; Thermoleophilia*
*Chlorella variabilis NC64A*	2507981240	Selenocysteine lyase	2508443779	*Saprolegnia parasitica CBS 223.65*	*Eukaryota; Oomycota; Saprolegniales*
*Volvox carteri f. nagariensis 69-1b*	2507997145	Selenocysteine lyase	2509158209	*Entamoeba histolytica HM-1:IMSS*	*Eukaryota; Evosea; Mastigamoebida*
*Chlamydomonas reinhardtii CC-503 cw92 mt+*	649484656	Selenophosphate synthase	2507499783	*Crocosphaera subtropica BH63E*	*Bacteria; Terrabacteria group; Cyanobacteria*
*Chlorella variabilis NC64A*	2507981440	Cobalamin synthesis protein cobW	640895065	*Roseiflexus castenholzii HLO8, DSM 13941*	*Bacteria; Chloroflexi; Chloroflexia*
*Chlamydomonas reinhardtii CC-503 cw92 mt+*	649491021	Cobalamin synthesis protein cobW	640596696	*Roseiflexus* sp. *RS-1*	*Bacteria; Chloroflexi; Chloroflexia*
*Physcomitrella patens patens*	649500991	Cobalamin synthesis protein cobW	649849043	*Pantoea* sp. *At-9b*	*Bacteria; Proteobacteria; Gammaproteobacteria*
Acid resistance					
*Sorghum bicolor BTx623*	2507715021	Spermidine/putrescine transporter	2509391205	*Rhizobium leguminosarum bv. trifolii WSM597*	*Bacteria; Proteobacteria;*
*Volvox carteri f. nagariensis 69-1b*	2508000101	Arginine deiminase ArcA (EC 3.5.3.6)	639619926	*Dictyostelium discoideum AX4*	*Eukaryota; Evosea; Eumycetozoa*
*Chlamydomonas reinhardtii CC-503 cw92 mt+*	649410730	Proton extrusion protein PcxA	640014237	*Lyngbya* sp. *PCC 8106*	*Bacteria; Cyanobacteria; Oscillatoriales*
*Chlamydomonas reinhardtii CC-503 cw92 mt+*	649494337	Arginine deiminase ArcA (EC 3.5.3.6)	649376570	*Naegleria gruberi NEG-M (Amoeba)*	*Eukaryota; Heterolobosea; Vahlkampfiidae*
*Chlorella variabilis NC64A*	2507979843	Carbamate kinase (EC 2.7.2.2)	643475399	*Rippkaea orientalis PCC 8801*	*Bacteria; Cyanobacteria; Chroococcales*
*Chlamydomonas reinhardtii CC-503 cw92 mt+*	649494190	Polyamine transporter 3	649064649	*Nannizzia gypsea CBS 118893*	*Eukaryota; Ascomycota; Eurotiomycetes*
*Physcomitrella patens patens*	649499876	Basic amino acid/polyamine antiporter, APA family/solute carrier family 7 (L-type amino acid transporter), member 9	639684312	*Candidatus Solibacter usitatus Ellin6076*	*Bacteria; Acidobacteria; Acidobacteriia*
*Physcomitrella patens patens*	649531109	Argininosuccinate lyase	651569762	*Paenibacillus* sp. *HGF7*	*Bacteria; Firmicutes; Bacilli*
*Ricinus communis Hale*	649573998	Spermidine/putrescine transport system	2508732231	*Microvirga lupini Lut6*	*Bacteria; Proteobacteria;*
*Ricinus communis Hale*	649590952	Argininosuccinate synthase (EC 6.3.4.5)	640876950	*Parvibaculum lavamentivorans DS-1*	*Bacteria; Proteobacteria; Alphaproteobacteria*

### HGT genes for metal resistance

The accumulation of toxic metals in contaminated soils stands for a serious environmental challenge, which negatively affects plant health and growth. As a possible solution, it has been reported that metal-resistant bacteria/fungi inhabiting plant-related sphere have the ability to promote plant growth and tolerance upon the attack of toxic metal stress (Mishra et al., 2017; Zhou et al., 2020), forming plant-microbe interactions which might also provide an opportunity for plants to acquire metal-resistant genes originated from nearby microbial taxa. In accordance, we identified eleven HGT gene entries for metal resistance in the tested plant genomes (**Table 2**), most of which are metal ion transport proteins (e.g., ChrA, CopA, CorA) that facilitate the efflux of cytosolic excessed toxic metal ions. Other HGT genes related to metal resistance might include phosphatase (**Table 2**), which might be secreted to increase soil phosphorus availability and immobilize toxic metal ions by forming complex compounds with them (Bechtaoui et al., 2021).

**Figure 1:**
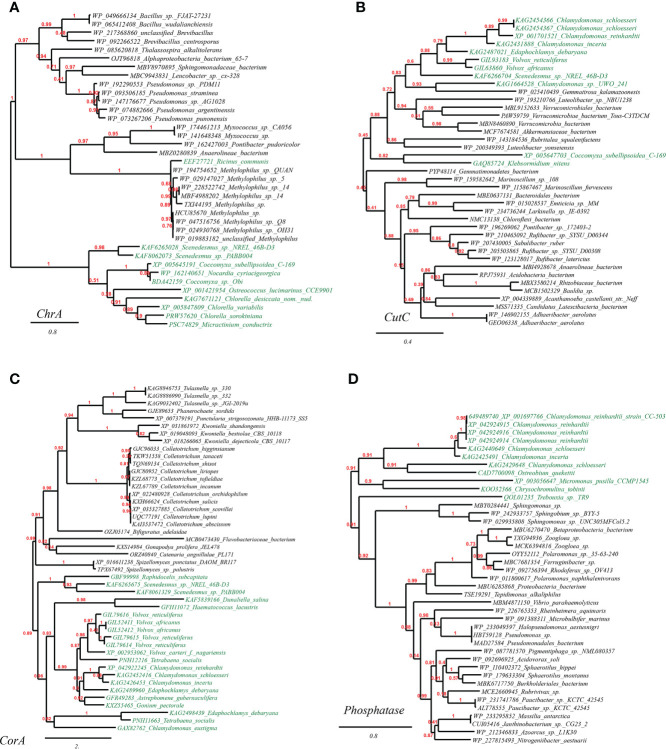
Phylogenetic tree of metal resistance proteins in plant genomes originated from microbial taxa. The phylogeny construction was conducted based on gene-translating protein sequences using PhyML program with the Maximum Likelihood (ML) method and 1,000 bootstrap replicates (with bootstrap values in red showing on respective branches). Branches leading to plant taxa are marked by green color, whereas those belonging to microbial lineages are in black: **(A)** chromate transporters (ChrA); **(B)** copper homeostasis protein (CutC); **(C)** magnesium and cobalt transport protein (CorA). **(D)** phosphatase.

To confirm the HGT origin of the mentioned plant metal resistance genes, we selected and built the phylogenetic trees with three representative genes as queries: chromate transporters (ChrA) of *Ricinus communis*, copper homeostasis protein (CutC) of *Chlamydomonas reinhardtii*, and magnesium and cobalt transport protein (CorA) of *Volvox carteri*. As expected, all these suggested HGT genes in plant genomes are clustering with homologues of cross-phylum microbial taxa (putative HGT donors). To be specific, it is illustrated in the well-supported phylogenetic tree constructed with not-exclusive top BLASTP hit entries that: ChrA of *Ricinus communis* (eudicots) and several green algae (e.g., *Chlorella* spp., *Chlamydomonas* spp., *Coccomyxa* spp.) bear significant resemblance to the homologues of vast *Proteobacteria* and *Firmicutes* lineages (e.g., *Methylophilus* spp., *Pseudomonas* spp., and *Bacillales*) (**Figure 1A**); CutC homologues of green algae (e.g., *Chlamydomonas* spp., *Volvox* spp., *Klebsormidium nitens*) are completely nested within in bacterial taxa (e.g., *Verrucomicrobiales, Bacteroidales, Rhizobiaceae*), a pattern indicative of cross-kingdom HGT (**Figure 1B**); besides, CorA homologues of green algae (e.g., *Chlamydomonas* spp., *Scenedesmus* spp., *Volvox* spp.) are closely clustering with fungal lineages (e.g., *Colletotrichum* spp., *Spizellomyces* spp., *Kwoniella* spp.) (**Figure 1C**). Lastly, phosphatase homologues of green algae (e.g., *Chlamydomonas* spp., *Ostreobium* spp., *Micromonas* spp.) are clustering adjacent to bacterial taxa (e.g., *Sphingomonas* spp., *Zoogloea* spp., *Pseudomonadales*), indicating strong HGT signals (**Figure 1D**).

### Osmotic and drought stress resistance

Salinization and drought of arable land stand for major challenges to agriculture production worldwide. Salt accumulation and water scarcity would cause osmotic and water-deficit stress that restrict plant growth by affecting nutrient uptake and cell physiology (Bashir et al., 2014; Hanin et al., 2016). Acquisitions of osmotic and drought stress resistance genes from nearby microbes to enhance self-resistance might be an alleviating approach for plants. Accordingly, we found ten gene entries that putatively confer osmotic stress resistance in five tested plant genomes (**Table 2**): eight ion/solute transporter (e.g., sodium/potassium ATPase, Na^+^/proline symporter, chloride channel protein and potassium/proton antiporter) and two organic osmolyte biosynthesis enzymes (choline dehydrogenase BetA and sarcosine oxidase SoxA). These genes are correspondent to two mechanisms of osmotic stress resistance: maintaining cellular ion homeostasis and recruitment of compatible solutes (e.g., proline, betaine, sarcosine) to stabilize proteins. These compatible solutes can also protect against drought, heat or cold stresses (Bashir et al., 2014; Hanin et al., 2016). Other putative HGT and osmotic/drought resistance genes detected in plants, as listed in **Table 2**, may include: trehalose 6-phosphate synthase and trehalose hydrolase, and dTDP-D-glucose 4,6-dehydratase (RmlB), involving in the biosynthesis of protective sugar; cellulose synthase, significant for enhanced cell wall (Behr et al., 2015). Besides, we have found in the plant genomes HGT-driven malate synthase and isocitrate lyase of the glyoxylate cycle, which were reported to be highly expressed under drought conditions (Todaka et al., 2015; Kim et al., 2020); Lastly, transaldolase, an upstream enzyme controlling erythritol (protectant) production, which was found up-regulated upon exposure to short-term drought stress (Liu et al., 2015) and hyper-osmotic stress (Iwata et al., 2015; Yang et al., 2015).

**Figure 2:**
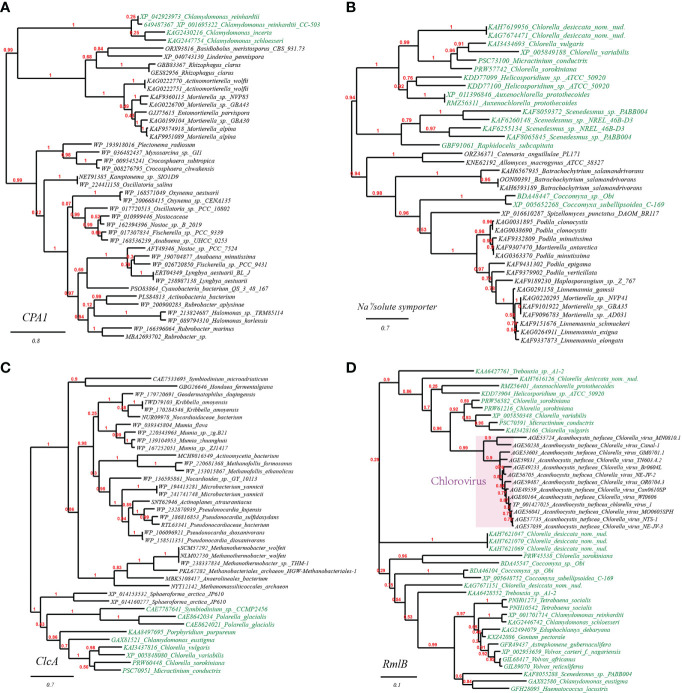
Phylogenetic tree of osmotic and drought stress resistance proteins in plant genomes originated from microbial taxa. The phylogeny construction was conducted based on gene-translating protein sequences using PhyML program with the Maximum Likelihood (ML) method and 1,000 bootstrap replicates (with bootstrap values in red showing on respective branches). Branches leading to plant taxa are marked by green color, whereas those belonging to microbial lineages are in black. **(A)** sodium/proton antiporter (CPA1); **(B)** Na^+^/solute symporter; **(C)** chloride channel protein (ClcA); **(D)** dTDP-D-glucose 4,6-dehydratase (RmlB).

To further verify the occurrence of HGT on the plant stress resistance genes mentioned above, we chose and built the phylogenetic trees of three representative genes: sodium/proton antiporter (CPA1) from *Chlamydomonas reinhardtii*, Na^+^/solute symporter from *Arabidopsis thaliana*, chloride channel protein (ClcA) and dTDP-D-glucose 4,6-dehydratase (RmlB) from *Chlorella variabilis* (**Figure 2**). As observed from the well-supported phylogenetic tree of CPA1, homologues from *Chlamydomonas* spp. are tightly clustering with CPA1 from fungi (e.g., *Basidiobolus, Rhizophagus, Actinomortierella*), and remotely with CPA1 from cyanobacteria and actinobacteria (**Figure 2A**). Likewise, the well-supported phylogenetic tree of Na^+^/solute symporter placed green algae/plant homologues (e.g., *Scenedesmus*, *Coccomyxa*, and *Klebsormidium*) in the vicinity of those of fungi (e.g., *Batrachochytrium, Podila, Mortierella*) (**Figure 2B**). Similarly, in the well-supported phylogenetic tree of ClcA (**Figure 2C**), homologues from *Chlorella* spp. (green algae) clustering with several homologues of other green algae (e.g., *Chlamydomonas*, *Micractinium*), are embedded in branches derived from archaeal taxa of phylum Euryarchaeota (*Methanobacteriales, Methanomassiliicoccales*) and high G+C Gram-positive bacterial taxa (e.g., *Pseudonocardia* spp., *Mumia* spp. and *Microbacterium* spp.), suggesting the occurrence of cross-phylum HGT. Finally, the phylogenetic tree of RmlB indicates that *Chlorella virus Acanthocystis turfacea* has facilitated the transitions of RmlB encoding genes (**Figure 2D**), in consistence with previous studies (Parakkottil et al., 2010).

### HGT genes in response to heat and cold stress

Large fluctuations in environmental temperature caused by inconstant global climate also render severe abiotic impacts (heat and cold stress) on plants. To cope with such conditions, gene inventions and expansions of heat/cold shock factor gene families into plant genomes are constantly happening during adaptive evolution (Wang et al., 2018; Li et al., 2021). In this study, we have also detected in tested plant genomes a wide range (26 gene entries) of microbial-originated HGT gene families related to heat/cold shock response (**Table 2**), which mainly function as transcription factors and molecular chaperones regulated by the former, working corporately to maintain cellular protein homeostasis (Andrási et al., 2021). In addition, it is reported that chitinase is also involved in plants’ cold and osmotic stress responses (Cao et al., 2019), and correspondingly, HGT gene entries encoding chitinases are also detected (**Table 2**). In addition, we have discovered HGT genes encoding ribulose 1,5-bisphosphate carboxylase large subunit (Rubisco), carbonic anhydrase (CA), and photosystem II proteins putatively acquired from Bacteria (**Table 2**). It is possible that plants might depend on generating more photosynthesis-related protein copies as a remedy strategy to cope with the impaired CO_2_-fixing efficiency resulted from malfunction of heat-liable Rubisco under heat-stress conditions (Nouri et al., 2015).

**Figure 3:**
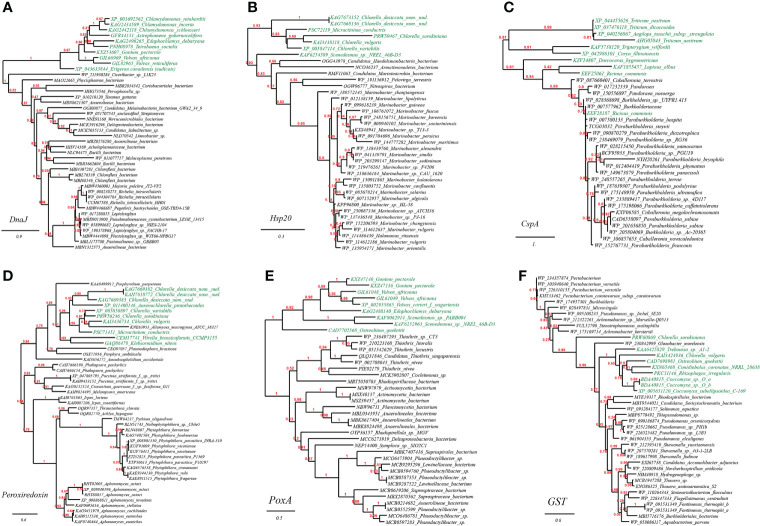
Phylogenetic tree of heat and cold stress as well as oxidative stress resistance proteins in plant genomes originated from microbial taxa. The phylogeny construction was conducted based on gene-translating protein sequences using PhyML program with the Maximum Likelihood (ML) method and 1,000 bootstrap replicates (with bootstrap values in red showing on respective branches). Branches leading to plant taxa are marked by green color, whereas those belonging to microbial lineages are in black: **(A)** heat-shock protein DnaJ; **(B)** heat shock protein Hsp20; **(C)** cold-shock protein CspA; **(D)** peroxiredoxin; **(E)** heme peroxidase (PoxA); **(F)** glutathione S-transferase (GST).

As before, we chose and performed phylogenetic analyses on several such genes to illustrate the likely evolutionary path. The first gene query is heat-shock protein DnaJ, which is reported to protect Rubisco activity under heat stress (Wang et al., 2015). As expected, we found that DnaJ proteins from a group of green algae (e.g., *Chlamydomonas*, *Tetrabaena, Edaphochlamys*, *Volvox*) and eudicots (*Erigeron canadensis*) (**Figure 3A**, marked with green color) are placed just next to homologues from bacterial lineages (e.g., *Phycisphaerae, Anaerolineae, Pseudanabaenaceae*). The second tested gene is heat shock protein Hsp20 (**Figure 3B**). Likewise, a group of green algae (e.g., *Chlorella*, *Micractinium, Scenedesmus*) is located in the well-supported tree of Hsp20 next to various bacterial taxa (e.g., *Marinobacter* spp., *Marinimicrobia*, *Nitrospirae*). The final case present the phylogeny of typical cold-shock protein CspA that promotes the correct folding of RNA molecules (Rennella et al., 2017). We found that several CspA-like proteins from eudicots (e.g., *Ricinus communis*, Lupinus albus) are in close homology to CspA proteins from *Betaproteobacteria* lineages (e.g., *Burkholderiaceae, Methylophilaceae*) (**Figure 3C**). Together, these phylogenetic relations might be existing molecular evidence supporting the HGT origins of plant abiotic resistance genes.

### HGT genes resisting oxidative stress

Oxidative stress resulting from the excessive generation of reactive oxygen species (ROS) that damage cellular biomolecules, including proteins, DNA, lipids, and carbohydrates, is the usual consequence of miscellaneous abiotic stresses in plants mentioned above (e.g., drought (Niu et al., 2021), osmotic pressure (Cai-Hong et al., 2005), toxic metal stress (Schützendübel and Polle, 2002)). As illustrated in our results (**Table 2**), it seems a potential strategy for plants to cope with the omnipresent oxidative stresses by obtaining directly from nearby microorganisms relevant resistance genes belonging to the canonical antioxidant machinery (e.g., hame peroxidase, glutaredoxin, thioredoxin, peroxin, peroxiredoxin, glutathione-S-transferase, cysteine synthase and glutamate/glutamine synthase) (Gill and Tuteja, 2010; Hasanuzzaman et al., 2019). Furthermore, miscellaneous HGT genes encoding components of oxidative electron transfer chain such as cytochrome oxidase (an oxygen-reducing reducing proton-pump) were also discovered (**Table 2**), which are suggested to be involved in oxidative stress and acid stress tolerance (de la Garza-García et al., 2021). In addition, also discovered in tested plant genomes were bacteria/fungi-originated genes encoding an isocitrate dehydrogenase (in *Arabidopsis thaliana*), which might supply reductant NADPH to defend against oxidative stress (Komatsu et al., 2014), a phytoene dehydrogenase (in *Chlorella variabilis*) that converts phytoene into antioxidant carotene, a homocysteine S-methyltransferase (in *Volvox carteri*) that generates antioxidant methionine, as well as genes related to biosynthesis of antioxidant vitamins thiamine and cobalamin in multiple plants (Rosado-Souza et al., 2020; Vásquez et al., 2022), as well as selenophosphate synthase and selenocysteine lyase putatively conferring oxidative stress protection (Costa et al., 2011) (**Table 2**).

As verified through phylogenetic analysis, the tested HGT genes in plants are all faithfully clustering with distant microbial taxa (the HGT donors), showing strong HGT signs (**Figure 3**). To be specific, in the well-supported phylogenetic tree of peroxiredoxin, homologues from the green algae (e.g., *Chlorella, Auxenochlorella, Micractinium*) and charophytic algae *Klebsormidium* are embedded in those from fungi (e.g., *Phakopsora, Puccinia, Irpex*), which indicated that the green algae might have gained peroxiredoxin genes through gene exchange from nearby fungi (**Figure 3D**); Similarly, as seen from the reconstructed phylogeny of heme peroxidase (PoxA), a group of green algae (e.g., *Gonium, Volvox, Scenedesmus, Ostreobium*) is placed in the vicinity of multiple bacterial lineages (e.g., *Thiothrix, Rhodospirillaceae, Anaerolineales*) (**Figure 3E**); Lastly, in the phylogeny of glutathione S-transferase (GST), homologues from the green algae (e.g., *Chlorella*, *Rhizophagus, Coccomyxa*) are enveloped by those from multiple bacterial lineages (e.g., *Pseudomonas, Achromobacter, Burkholderiales*), again, suggesting the cross-kingdom HGT of GST genes among such taxa (**Figure 3F**).

### HGT genes contributed to pH homeostasis

Environmental pH is considered as an important factor for plants since it influences soil physical, chemical properties and nutrient availability and thereby affects plant inner homeostasis and growth (Zhou et al., 2022). Interestingly, we have excavated seven microbe-derived gene entries linking with pH homeostasis maintenance and acid stress resistance in five tested plant genomes (**Table 2**): two basic amino acid/spermidine/putrescine transporter and a proton extrusion protein (PcxA) encoding genes acquired from Bacteria, and genes within the arginine deiminase pathway (argininosuccinate synthase, argininosuccinate lyase, carbamate kinase and arginine deiminase) derived from Bacteria and Amoeba, which might conduct pH regulation by neutralization of proton with base substrates like ammonia, arginine and polyamine. Besides, the mentioned osmotic stress resistance ion/proton transporter above might also take part in adaptive responses to pH perturbation.

**Figure 4:**
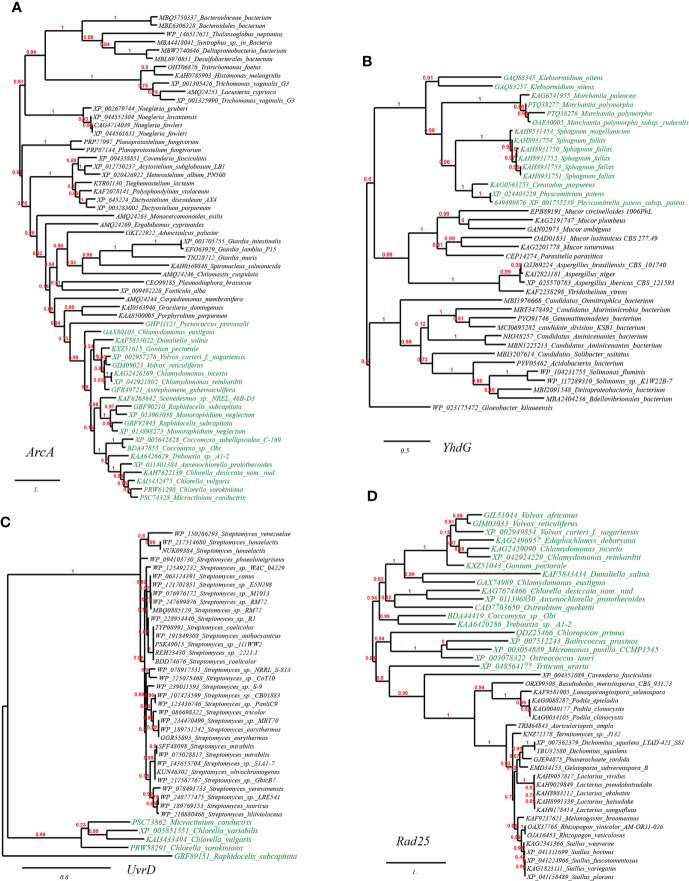
Phylogenetic tree of acid resistance and DNA damage resistance proteins in plant genomes originated from microbial taxa. The phylogeny construction was conducted based on gene-translating protein sequences using PhyML program with the Maximum Likelihood (ML) method and 1,000 bootstrap replicates (with bootstrap values in red showing on respective branches). Branches leading to plant taxa are marked by green color, whereas those belonging to microbial lineages are in black. **(A)** arginine deiminase (ArcA); **(B)** basic amino acid/polyamine antiporter (YhdG); **(C)** chloride channel protein (ClcA); **(D)** dTDP-D-glucose 4,6-dehydratase (RmlB).

We further selected and constructed the phylogeny of two representative HGT genes, which encode arginine deiminase (ArcA) in *Volvox carteri* (chlorophyte algae) and basic amino acid/polyamine antiporter (YhdG) in *Physcomitrella patens* (mosses), to test the reliability of the HGT inference (**Figure 4**). Consistency, the well-supported phylogenetic tree constructed with not-exclusive top hit entries showed that ArcA homologues of *Volvox* spp. are clustering with those of several green algae affiliations like *Scenedesmus* spp., *Chlamydomonas* spp., and *Chlorella* spp. etc. (**Figure 4A**, marked with green color), which are wholly embedded in homologous sequences from protist (e.g., *Carpediemonas, Fonticula, Plasmodiophora* and *Naegleria* etc.), the most likely cross-phylum HGT donor(s), and also, remotely associated with a cluster of Bacteria (e.g., *Desulfobacterales*). Likewise, the well-supported phylogenetic tree of YhdG showed that homologues of *Physcomitrella*, closely clustering with homologues of other mosses (e.g., *Sphagnum* spp., *Ceratodon purpureus*), liverworts (*Marchantia* spp.), and a green plant (*Klebsormidium nitens*), are nested within homologues of fungi (e.g., *Mucor* spp. and *Aspergillus* spp.) and bacteria (e.g., *Acidobacteria*, *Aminicenantes* and *Solimonas*), strongly indicating that cross-phylum HGTs might contribute to the spread of YhdG from microbes to plants (**Figure 4B**).

### HGT genes contribute to ultraviolet light damage resistance

The plant DNA can be damaged by various abiotic factors such as chemical mutagens or ultraviolet (UV) light radiation due to long-term sun exposure, which would induce oxidative damage and cross-links (DNA–protein or DNA–DNA). The accumulation of such damages in the plant would ultimately cause genomic instability and cell death (Tuteja et al., 2009). Apart from cellular DNA-damage response pathways encoded by plants themselves, our results suggested that many plants have also recruited plenty of genes with functions related to DNA repair/replication/recombination (68 gene entries, see **Table S1A** at https://doi.org/10.6084/m9.figshare.20530083.v1). For example, we have also found in the reconstructed phylogeny of UvrD, a DNA helicase involved in excision repair of UV-induced DNA damage, that green algae homologues (e.g., *Micractinium*, *Chlorella*, *Raphidocelis*) are placed in the vicinity of homologues from cross-phylum *Streptomyces* spp. (fungi), which indicated the occurrence of HGT event (**Figure 4C**); Likewise, we found that several green algae (e.g., *Volvox*, *Chlorella*, *Chlamydomonas*) contain Rad25, a DNA helicase required for DNA repair and RNA polymerase II transcription, which is in close sequence homology to that from fungi (e.g., *Podila, Lactarius, Auriculariopsis*) (**Figure 4D**).

### HGT genes contribute to organic pollutant resistance

Organic soil pollution discharged by anthropogenic processes like herbicides overuse and chemical waste mismanagement can threaten the life of plants, and the association of endophytic or mycorrhizal microbes with host plants can reduce the harmful effect of organic pollutants to plant (Sun et al., 2015; Rajtor and Piotrowska-Seget, 2016). Interestingly, we found that plants have integrated various enzymes related to organic pollutant catabolism and several multidrug transport proteins into their genomes, such as cytochrome P450s, laccase, beta-lactamase, alkanesulfonate monooxygenase, haloperoxidase, most likely through HGT from nearby microbes (81 gene entries, see **Table S1B** at https://doi.org/10.6084/m9.figshare.20530083.v1). The most commonly-seen HGT enzyme of these, cytochrome P450 (CytP450, six gene entries), is a group of oxygenase enzymes that can catalyze the catabolism of a wide range of organic pollutants using oxygen as the electron acceptor, undergoing various detoxification reaction, including hydroxylation, N-, O-, S-dealkylation, sulfurization, epoxidation, deamination, desulfurization, dehalogenation, peroxidation, and N- oxide reduction (Lin et al., 2022). In addition to microbial CytP450, two laccases for removal of phenolic pollutants (Asadgol et al., 2014) were found in the genomes of *Chlamydomonas reinhardtii* and *Arabidopsis thaliana*, respectively, putatively acquired from fungi. While other organic degradation oxidases we found acquired by plants from microorganisms might target more specific organic compounds, such as alkanesulfonate, amine and beta-lactams (see **Table S1B** at https://doi.org/10.6084/m9.figshare.20530083.v1).

**Figure 5:**
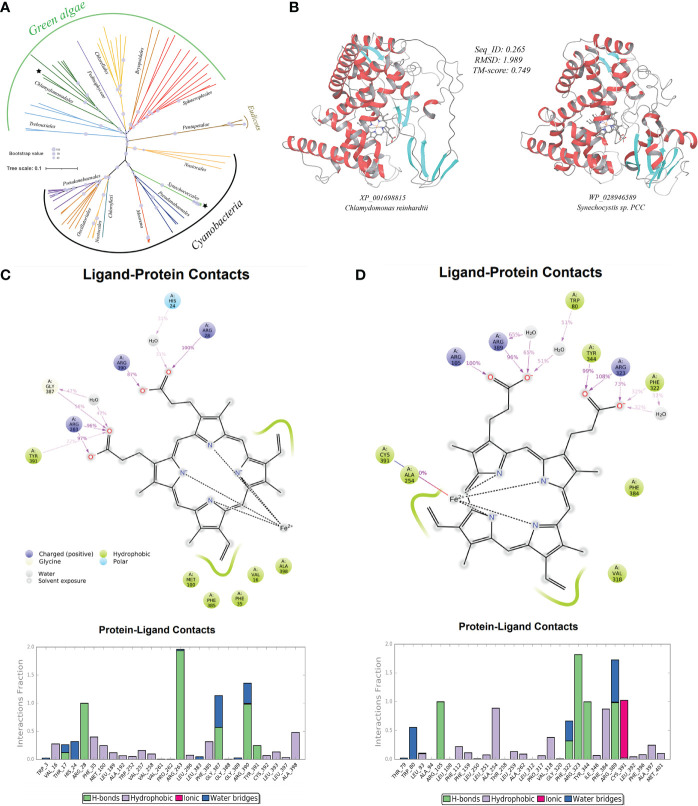
A case-study of microbe-originated plant cytochrome P450 and its donor protein. **(A)** Phylogenetic tree of the organic pollutant catabolism protein cytochrome P450 constructed by PhyML program with the Maximum Likelihood (ML) method and 1,000 bootstrap replicates (with bootstrap values shown by size of symbols); **(B)** structure perdition (deep-learning-driven) and comparison of representative recipient plant cytochrome P450 (left, *Chlamydomonas reinhardtii*: XP_001698815) and its putative microbial donor (right, *Synechocystis* sp. PCC: WP_028946589); **(C)** ligand-protein simulation diagram (5 ns classical molecular dynamics) of cytochrome P450 from *Chlamydomonas reinhardtii* (XP_001698815) showing key residues in proteins interacting with the hame prothetic group (ligand). Interactions that occur more than 30.0% of the simulation time in the selected trajectory (0.0 through 5.0 ns), are shown; **(D)** ligand-protein simulation diagram (5 ns classical molecular dynamics) of the cytochrome P450 from donor *Synechocystis* sp. PCC (WP_028946589) showing key residues in proteins interacting with the hame prothetic group (ligand) for comparison.

As before, the HGT possibility was tested on representative genes. In the well-supported phylogenetic tree using CytP450 from *Chlamydomonas reinhardtii* (XP_001698815) as query and constructed with not-exclusive top 100 BLASTP hit entries showed that CytP450 homologues of green algae (e.g., *Chlamydomonadales, Trebouxiales, Chlorellales, Sphaeropleales*), along with several homologues from eudicots (*Pentapetalae*), are placed in the neighborhood of homologues from cyanobacteria (e.g., *Synechococcales, Nostocales, Pseudanabaenales*), clearly indicating the occurrence of cross-kingdom HGT events that have facilitated the acquisitions and spread of organic pollutant catabolism enzyme CytP450 from lower-grade microbial lineages to various plant taxa (**Figure 5A**).

### Assessment of the functionality of HGT genes

To assess if the above-mentioned abiotic resistance genes in plants are functioning normally in plants, we resorted to protein structure analysis methods to assess fold reasonability on one hand and codon adaption index (CAI) calculation for prediction on gene expression potential of the abiotic stress resistance genes on the other hand.

We first tested if proteins encoded by such HGT-originated abiotic stress resistance genes in plant genomes can fold themselves into reasonable three-dimensional (3D) structures conveying acknowledged molecular functions. With the aid of the excellent deep-learning-driven structure prediction programs, AlphaFold2 (Jumper et al., 2021) and RoseTTAFold (Baek et al., 2021), we have generated full-length structure predictions of proteins encoded by all detected microbe-originated HGT genes in plants related to abiotic stress resistance in this study. The outcoming models of both methods in this test are available at https://doi.org/10.6084/m9.figshare.20530026.v1. The TM-align-based comparison showed that the predicted models from both methods (AlphaFold2 and RoseTTAFold) agreed well with each other, giving an average pairwise TM-score of 0.80 (TM-score > 0.5 indicates similar fold), indicating that our protein structural models are very reliable. We further used such protein models as inputs of the deep-learning-driven structure-based protein functional annotation program DeepFRI (Gligorijević et al., 2021) for functional assessment. Results showed that about 68.5% molecular function annotations of these HGT-driven proteins have reliable confident scores above the DeepFRI significance cutoff score of 0.5 (see **Table S1C** at https://doi.org/10.6084/m9.figshare.20530083.v1), strongly indicating that most of the aforementioned HGT-originated abiotic stress resistance proteins can fold themselves into a correct, recognizable, and functional 3D topology. Furthermore, TM-align-based structural alignments of the 3D models of protein homologue from corresponding microbial donors built by the same deep-learning method (The outcoming models are available at https://doi.org/10.6084/m9.figshare.20530026.v1) to the plant HGT-driven abiotic stress resistance proteins also reveal similar fold with a relatively high average pairwise TM-score of 0.72 (see **Table S1D** at https://doi.org/10.6084/m9.figshare.20530083.v1). To give more impression, we provided here a case study of the above-mentioned CytP450 from *Chlamydomonas reinhardtii* (**Figure 5B**). Comparisons of the CytP450 protein structure from the plant representative with that from the predicted microbial donor representative (*Synechocystis* sp. PCC) reveal a pair of folds showing highly identical topology with pairwise TM-score of 0.75 and average root mean square deviations (RMSD) of 1.99 Angstrom (Å). Resemblance was further found as we compared ligand-protein interaction patterns of these two structures by conducting classical molecular dynamics for 5 ns (**Figures 5C, D**). Results showed that both proteins used conserved residues like positively charged Arg and aromatic Tyr, Phe for ligand interactions throughout the simulation, suggesting strong evolutionary connection and reaction feasibility. Similar fold resemblances were found in structures derived from other pairs of HGT genes (see **Figure S1** at https://doi.org/10.6084/m9.figshare.21008905.v1).

**Figure 6:**
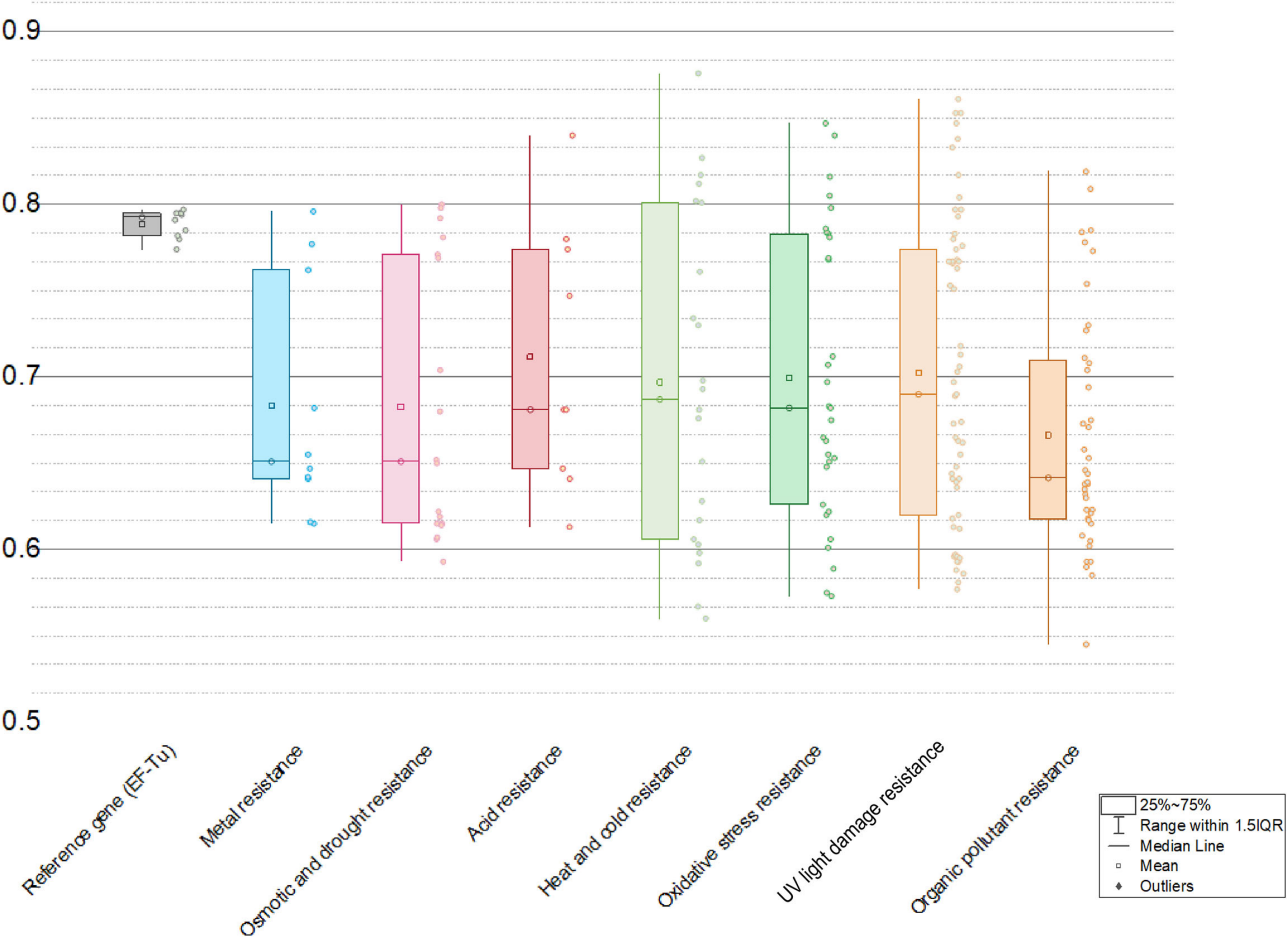
Ranges of codon adaption index (CAI) values of different kinds of abiotic resistance genes with EF-Tu gene as a reference.

Due to translational selection, genes with high expression level generally have a stronger codon bias than those expressed at lower levels (Hiraoka et al., 2009; Zhou et al., 2013). The codon adaption index (CAI) initially proposed by Sharp and Li (1987) can measure synonymous codon usage bias for a tested gene sequence by comparing the similarity between the synonymous codon usage of a gene and the synonymous codon frequency of validated highly expressed reference gene sets. CAI value ranges from 0 and 1, with higher value indicating higher synonymous codon usage similarity with the reference/higher expression level (Puigbò et al., 2008). The CAI method has been successfully used in various areas, such as the predictions on expression likelihood (Puigbò et al., 2007; Puigbò et al., 2008) and level (Li et al., 2019) of heterologous/HGT gene, and deduction of lifestyles based on genomic data (Willenbrock et al., 2006). In this study, the expression levels of all predicted HGT genes conferring abiotic stress resistance in the plant genomes were assessed utilizing the codon adaption index (CAI) as a numerical estimator. Results showed that many HGT-driven abiotic stress resistance genes in plant genomes possess relatively high CAI values (**Figure 6**): averagely, metal resistance (0.683), osmotic and drought stress resistance (0.682), acid resistance (0.712), heat and cold stress resistance (0.697), UV light damage resistance (0.702), and organic pollutant resistance (0.681). The HGT genes related with pH homeostasis and DNA repair have relative higher CAI values, which might indicate that the pH turbulence and UV light radiation are the most commonly encountered abiotic stresses.

Together, it means that the strategy of acquisitions of abiotic stress resistance genes by plants from microbes to enhance adaption might be successful and significant as far as our results corroborate.

## Discussion

In this study, we have discovered an abundant repertoire of putative microbial-originated genes in 14 tested plant genomes that confer resistance to a wide range of abiotic stresses commonly encountered by plants, with the putative microbial HGT donors spanning from viruses, bacteria, fungi to protists (**Table 2** and see **Table S1** at https://doi.org/10.6084/m9.figshare.20530083.v1). We have also applied in-silico approaches to verify the accuracy of blast-based HGT inference (phylogenetic analyses), the functionality of the 3D protein fold encoded by such HGT genes (deep-learning-driven protein structure prediction, fold alignment, molecular dynamics etc.), and the expression potential of HGT genes (codon adaption index calculation).

The emergence and mechanism of HGT events (“Why does lateral transfer occur in so many species and how?”) is recommended as a still-pending and significant scientific question worthy of more in-depth ivestigations by the editorial of the journal Science in the article “So much more to know” (American Association for the Advancement of Science, 2005), and we prudently considered that the present research might have supplied some hints for this question. HGT can serve as a significant force that supplies species with genetic innovations and regulation flexibility that guarantees successful migration into novel niches. In our results, we found that the most commonly seen microbial HGT donor taxon for acquisition of resistance genes is *Burkholderiaceae* (n=10). Members of *Burkholderiaceae* are characterized as plant-associated, which usually constitute the core floral community (Massoni et al., 2021). Besides, we found that *Chlorophyta* taxa (e.g. *Chlorella variabilis* NC64A, n=42) generally have more HGT resistance genes than lower-grade *Streptophyta* taxa (e.g. *Physcomitrella patens patens* (moss), n=21), which outnumber those from high-grade land plant (e.g. *Ricinus communis Hale* (castor bean), n=14). Such richness of HGT genes associated with abiotic stress resistance especially those within the transition-stage lineages might have reflected a ring linking to the early adaption processes of plant to accomplish the colonization of land environment from aquatic. These HGT genes seem well-suited to combat abiotic factors more frequently encountered in land environments (e.g., desiccation, fluctuations of temperature, UV radiation), in consistence with previous studies (de Vries et al., 2016; Pennisi, 2019; Bowles et al., 2020). The mechanism leading to such abundant cross-lineage gene transfer into plant genomes might be explained by the “weak-link model” (Huang, 2013). Namely, foreign DNA fragments (e.g., from microbes) could be naturally imported into the plant receptors at the susceptible stages in plant lifecycle, such as the single-cell/preliminary stages (germline cells, embryos, etc.) or dedifferentiation/asexual reproduction stages. The microbe-plant HGT processes can be promoted by intimate or direct physical contact through symbiosis, parasitism, infection, or other associations, especially when the weakly-protected cells are exposed to the environment. Once getting into the cell, fixation of HGT genes to the recipient genome can be facilitated by the still undergoing organellar DNA fragment integrations into the nuclear chromosome in the developing cells, followed by vertical transfer of integrated HGT genes to offspring cells *via* mitosis (Huang, 2013).

Concerns regarding such cross-lineage HGT genes might be the authenticity of both HGT direction and identification during the inference and the functionality of such HGT genes in the plant genomes. It has been argued that some of such HGT genes in the eukaryotic genome might result from endosymbiotic origin or contamination (Koutsovoulos et al., 2016).

To alleviate these concerns, we have integrated into our study the “gold standard” verification for the detected HGT genes: that a particular gene has been gained through HGT from a different lineage would be phylogenetic incongruence, where an evolutionary tree for a specific protein family is distinct from the established organismal phylogeny (Schönknecht et al., 2014). For example, the well-supported phylogenetic tree of chromate transporters (ChrA) (Fig. 1a) shows the protein sequence from *Ricinus communis* and green algae embedded within bacterial sequences. The most parsimonious explanation for this phylogenetic distribution is HGT from bacteria. Regarding the direction of the gene acquisition (microbe to plant instead of the reverse), it is ensured through the HGT detection pipeline IMGAP v.5.0 that only selects HGT candidate genes showing the best hits outside the taxonomic lineage of the tested genome (i. e. from distant phylum, class, etc.), but with lower-scoring or no hits within the lineage of the tested genome. The precise method for identification of HGTs by the IMGAP pipeline has been expatiated by Markowitz et al. (2010).

Furthermore, our supplemented phylogenetic analyses have also supported the direction of the predicted HGTs. As for excluding the possibility of false positive induced by contamination, we included in our analyses only high-quality complete (or near-complete) genome assemblies of plants (**Table 1**), and our phylogenetic analyses further indicate that the historical HGT events not only occurred once but also can be confidently identified in cases in related plant taxa. For example, in the well-supported phylogenetic tree of copper homeostasis protein (CutC), the HGT-driven homologues were detected in a range of different green algae (from *Chlamydomonas* on the top of the tree to *Coccomyxa* inserted in the middle of the tree), again intertwined with branches leading to bacteria taxa. This suggested that such HGT events should be very likely actual since the probability that the said green algae’s genome sequences happen to contaminate simultaneously the same kind of DNA fragments in lab is minor.

As for the functionality of the HGT genes in the plant genomes, we have resorted to multiple methods (e.g., structure analysis and codon adaption index calculation) to illustrate the functionality of the HGT genes related to plant abiotic stress resistance. In line with our results, previous studies have experimentally verified that many horizontally transferred genes in plants *Orobanchaceae, Cuscuta* and *Rafflesia* are being expressed (Xi et al., 2012; Yang et al., 2016; Vogel et al., 2018; Yang et al., 2019). What’s even more surprising is that twelve of the HGT genes in *Alloteropsis semialata* (grass) genome have a higher expression level than their native homologues, and in one case, the native copy was replaced by the foreign one (Dunning et al., 2019). In consistence, our CAI calculation results also supported relatively high expression levels across the microbe-originated abiotic stress resistance genes in plants, which emphasized their significant functions in supporting the growth of the plant host in the face of harsh abiotic conditions.

In summary, the microbe-originated HGT genes in plant genomes identified in our analyses and their participation in resistance against diverse abiotic pressures commonly encountered in land environments indicate a widespread and profound impact of HGT on the evolution of plants and other eukaryotes. Still, our analysis presented here is not exhaustive. We believe that the microbe-to-plant HGT cases discovered by far stand for only the tip of the profound evolutionary iceberg. As more and more genomic sequences of superior quality become available for wider lineage branches in the tree of plants, it is foreseeable that future research will provide us with a more exciting panorama illustrating both the extent and the evolutionary significance of HGT in plants thoroughly.

## Materials and methods

Fourteen high-quality eukaryotic plant genomes covering major plant taxa (clade *Viridiplantae*) within phyla *Chlorophyta* and *Streptophyta*, as listed in **Table 1** were chosen and downloaded from the public database (Genbank/IMG) for downstream analyses. Identification of horizontally transferred genes in the genomes of plant genomes was conducted through the Integrated Microbial Genomes Annotation Pipeline (IMGAP) v.5.0 (Markowitz et al., 2010), which defined genes in tested plant genomes as having been horizontally transferred from a distant lineage with the principle: genes that have the best BLASTP hits (highest bit scores) or >90% of the best hits found outside the taxonomic lineage of the tested genome (i.e., from distant phylum, class, etc.) and with lower-scoring hits or no hits within the lineage.

The phylogeny of different kinds of abiotic resistance gene was constructed based on gene-translating protein sequences using PhyML program (Guindon et al., 2010) at http://www.phylogeny.fr/simple_phylogeny.cgi with the Maximum Likelihood (ML) method and 1,000 bootstrap replicates (with bootstrap values in red showing on respective branches) and visualized with iTOL (Letunic and Bork, 2021) at https://itol.embl.de/. Sequences were aligned with MUSCLE (Edgar, 2004) and trimmed with Gblocks (Talavera and Castresana, 2007) prior to tree construction.

AlphaFold2 (Jumper et al., 2021) and RoseTTAFold (Baek et al., 2021) were applied to generate the full-length 3D structure of proteins encoded by targeted HGT genes in plant genomes and their suggested donors, following guidance from https://github.com/deepmind/alphafold and https://github.com/RosettaCommons/RoseTTAFold. Docking of ligands into respective protein structure was conducted with AutoDock Vina v.1.2.0 (Trott and Olson, 2010). Molecular dynamics (MD) simulations for the targeted protein-ligand complex were performed using the Desmond Molecular Dynamics System, version 3.6, (D. E. Shaw Research, New York, NY, 2008) with OPLS_2005 force field (with default parameters) and visualized by Visual Molecular Dynamics (VMD) software v.1.9.4 and LigPlot+ (Laskowski and Swindells, 2011). DeepFRI (Gligorijević et al., 2021) was further applied for reliable structure-based functional annotation verify recognizable functional folds in the proteins encoded by the HGT genes related to abiotic pressure resistance in plant genomes.

Codon adaption index (CAI) was used as a numerical estimator of gene expression level (Hiraoka et al., 2009; Zhou et al., 2013), and correspondingly, the webserver CAIcal (Puigbò et al., 2008) (http://genomes.urv.es/CAIcal/calc.php) was applied to calculate respective CAI values of HGT genes in plant genomes participating in resistance of diverse abiotic pressures with the EF-Tu gene (the elongation factors) as refence, which is supposed to be highly expressed across most organisms (Puigbò et al., 2008), as conducted previously (Li et al., 2019). Besides, the Origin Pro 2020 software (OriginLab, Northampton, MA, USA) was used for data analysis and figure creation.

## Data availability statement

The data presented in the study are available in the Genbank and IMG repositories under accession numbers provided in **Table 1**.

## Author contributions

LL, and HY conceived and designed the research. LL, SP, ZW, TZ, HL, YX, JL and YL analyzed the data. LL wrote the manuscript. All authors contributed to the article and approved the submitted version.

## Funding

This research was supported by the key project of Science and Technology of Hunan Branch of China National Tobacco Corporation (202104, XX2022-2024Aa01, HN2021KJ05, 20-22A02, HN2020KJ02), the key research and development program of Hunan Province (grants no. 2020WK2022, 2022SK2076), the National Natural Science Foundation of China (NSFC) (No. 41807332), Fundamental Research Funds for the Central Universities of Central South University (no. 2022ZZTS0420) and Hunan International Scientific and Technological Cooperation Base of Environmental Microbiome and Application (No. 2018WK4019).

## Acknowledgments

We are grateful for resources from the High-Performance Computing Center of Central South University.

## Conflict of interest

Author ZW is employed by Zhangjiajie Tobacco Company of Hunan Province. Author TZ is employed by Hunan Urban and Rural Environmental Construction Co., Ltd. Authors YX and JL are employed by Chenzhou Tobacco Company of Hunan Province.

The remaining authors declare that the research was conducted in the absence of any commercial or financial relationships that could be construed as a potential conflict of interest.

The authors declare that this study received funding from Science and Technology of Hunan Branch of China National Tobacco Corporation. The funder was not involved in the study design, collection, analysis, interpretation of data, the writing of this article, or the decision to submit it for publication.

## Publisher’s note

All claims expressed in this article are solely those of the authors and do not necessarily represent those of their affiliated organizations, or those of the publisher, the editors and the reviewers. Any product that may be evaluated in this article, or claim that may be made by its manufacturer, is not guaranteed or endorsed by the publisher.
